# Neuroprotective Effects of MicroRNA-210 on Hypoxic-Ischemic Encephalopathy

**DOI:** 10.1155/2013/350419

**Published:** 2013-09-09

**Authors:** Jie Qiu, Xiao-yu Zhou, Xiao-guang Zhou, Rui Cheng, Hai-ying Liu, Yong Li

**Affiliations:** Department of Newborn Infants, Nanjing Children's Hospital of Nanjing Medical University, Nanjing 210008, China

## Abstract

*Objectives*. To reveal the effect of microRNA-210 on cell apoptosis caused by HIE. *Methods*. Postnatal day 7 rats after HI injury were intraventricularly injected with microRNA-210 mimic, microRNA-210 inhibitor, or physiological saline. 72 h after the injection, rats were sacrificed and the left hemispheres were collected. The expression level of microRNA-210 was identified by quantitative real-time PCR analysis. Apoptosis in brain sections was investigated by TUNEL assay. Apoptosis-related protein expressions were studied by Western blot analysis. *Results*. The results showed that microRNA-210, whose expression was downregulated in the brain 72 h after HI injury, suppressed neuronal apoptosis by inhibiting caspase activity and regulating the balance between bcl-2 and bax levels. *Discussion*. Recent study demonstrated that microRNA-210 has neuroprotective effects through inhibiting apoptosis in a murine model of HIE. It represents a potential novel therapeutic approach for the treatment of HIE.

## 1. Introduction

Hypoxic-ischemic encephalopathy (HIE) is the most important cause of cerebral damage and long-term neurological sequelae in the perinatal period both in term and preterm infants. Following HIE, approximately 45% of newborns die or have permanent neurological deficits including cerebral palsy, mental retardation, and epilepsy, and there are currently no effective therapies. 

The developmental susceptibility of the brain after hypoxic-ischemic (HI) injury is likely to be related to the transcriptional factor hypoxia inducible factor-1*α* (HIF-1*α*), a key regulator in the pathophysiological response to the stress of hypoxia, which plays a pivotal role in the development of injury and in normal brain development. Recently, microRNAs (miRs), which are small (18–25 nts) noncoding RNAs that regulate posttranscriptional gene expression by blocking translation of target mRNAs or by accelerating their degradation, have been reported to be induced by hypoxia. In particular, microRNA-210 (miR-210), which is activated by HIF-1*α* for the hypoxic induction [1], is a unique miR that is evolutionarily conserved and ubiquitously expressed in hypoxic cell and tissue types [2]. While miR-210 was initially thought to be intergenic, a more recent study showed that it is in fact contained within the sequence of a transcript with virtually unknown own function (AK123483) [3], which is also hypoxia inducible. MiR-210 plays multiple crucial roles in the cellular regulation in response to low oxygen including ischemic brain injury. Recent study suggested that miR-210 was expressed in both brain and blood of rat middle cerebral artery occlusion (MCAO) model [4, 5]. Furthermore, expression of miR-210 in human umbilical vein endothelial cells (HUVEC) results in increased tubulogenesis and increased vascular endothelial growth factor (VEGF)-induced cell migration through the repression of the receptor tyrosine kinase ligand Ephrin-A3 [6]. So, we hypothesized that miR-210 may have essential functions in HIE despite its role during HIE is not understood. 

Although proteins represent the overwhelming majority of therapeutic targets, recent developments of miR derivatives such as anti-miR oligonucleotides (AMOs) and locked nucleic acids (LNAs) are regarded as important steps toward clinical applications [7–9]. While there are many challenges for miRs as therapeutic targets such as delivery, potential off-target effects, and safety, the strategy of miRs manipulation *in vivo* to regulate disease-related processes is already becoming a feasible future therapeutic approach. This provides a potential new approach for treating a variety of diseases including cancer, viral infections, and cardiovascular and muscle diseases. Recently, Hu et al. demonstrated that delivery of miR-210 through a nonviral minicircle vector in the ischemic heart can improve heart function by promoting angiogenesis and inhibiting apoptosis [10]. Therefore, we hypothesized that miR-210 may lead to a novel therapy for HIE and showed the relationship between miR-210 and HIE in this study. 

## 2. Materials and Methods

### 2.1. Animal Models

This study was approved by the Institutional Animal Care and Use Committee of Nanjing Medical University. Pregnant Sprague-Dawley (SD) rats were housed in individual cages with 12 h light/dark cycles at 22 ± 2°C with free access to food and water. After normal delivery, the size of the litter was adjusted to 10 male rat pups to eliminate the gender difference of neonatal HIE. The mouse model for neonatal HIE was produced according to the methods reported previously in the literature [11] with minor modifications. Briefly, a less than 1 cm longitudinal midline incision of the neck was performed under ether anesthesia on postnatal day 7 (P7) rats. The left carotid artery was exposed and ligated permanently with a double suture. The entire surgical procedure lasted no longer than 10 min. Animals with excessive bleeding were excluded. The rat pups were returned to home cage with their dam for 1 h followed by exposure to hypoxia (92% N_2_ + 8% O_2_) for 2 h by placing them in an airtight chamber partially submersed in a 37°C water bath. At the end of 2 h hypoxia, the pups were returned to their dam again for recovery. The control animals received sham operation that consisted of left carotid artery exposure without ligation and then exposed to hypoxia for 2 h.

### 2.2. Intraventricular Injection

Rats were anesthetized with a mixture of ketamine (60 mg/kg) and xylazine (10 mg/kg) and then were placed in a stereotaxic apparatus (Stoelting, Wood Dale, IL, USA). Reagents including miR-210 minic (2.5 mg/kg), miR-210 inhibitor (2.5 mg/kg), or physiological saline (2.5 mg/kg) were injected with a microosmotic pump (Alzet 1007D; Durect Corp, Cupertino, CA, USA) into the lesioned side (left side) of the lateral ventricle (coordinates: anterior/posterior −0.9 mm, left 1.5 mm relative to bregma, and dorsoventral −5.0 mm from the dural surface). The injection was completed within 5 min, at speed 0.2 *μ*L/min; the glass pipette was kept in the position for an additional 2 min following injection and then slowly extracted. The rat pups were returned to home cage again. 72 h after the injection, rats were sacrificed either by cervical dislocation or decapitation for brain tissue preparation. The brains were divided into ipsilateral and contralateral hemispheres. The left hemispheres were collected and stored at −20°C until use. 

### 2.3. miRNA Real-Time Quantitative PCR

Total RNA (plus miRNA) was extracted from brain samples with use of the mirVana miRNA isolation kit from Ambion (Austin, TX, USA), according to the manufacturer's protocol. The concentration and integrity of RNA were determined by NanoDrop ND-1000 spectrophotometry (NanoDrop Tech, Rockland, Del) and gel electrophoresis, respectively.

Using a specific miR-210 and endogenous control U6 stem-loop primer, reverse transcription was performed according to the manufacture's protocol of the TaqMan miRNA RT Kit (Applied Biosystems, Foster City, USA). A total RNA (10 ng) was reverse transcripted to cDNA with 1 mM dNTPs (with dTTP), 50 U reverse transcriptase 1 *μ*L, 4 U RNase inhibitor in the presence of specific miR-210 or U6 stem loop reverse transcriptase primers in a 15 *μ*L system buffered by RT Buffer and DEPC water; following the thermal cycle program of 16°C for 30 min, 42°C for 30 min, and 85°C for 5 min, cDNA was stored at −20°C.

The real-time quantitative PCR was performed by a fast real-time PCR system (7900HT, ABI, USA) using a TaqMan miRNA assay kit. The reaction volume is 20 *μ*L containing the components as listed: miR-210 or U6 RT reaction product (1.33 *μ*L), 20×TaqMan MicroRNA assay (miR-210 or U6) 1 *μ*L, TaqMan 2×universal PCR master mix 10 *μ*L, and DEPC water 7.67 *μ*L. A 96-well plate was then run following the protocol as 95°C for 10 min, followed by 43 cycles of 95°C for 15 sec and 60°C for 1 min. Finally, the relative miR-210 level was normalized to the endogenous control U6 expression for each sample in triplicate and was calculated by the 2^−ΔCt^ method.

### 2.4. Terminal Deoxynucleotidyl Transferase-Mediated Uridine 5′-Triphosphate-Biotin Nick End Labeling (TUNEL) Staining

Brains were placed into 10% formalin immediately after excision and immersed for 24 h. Brain specimens were then embedded in paraffin and sections were cut at 5 *μ*m. The slides were incubated with 20 *μ*g/mL proteinase K for 15 min, rinsed with phosphate buffered saline (PBS), incubated with 3% H_2_O_2_ and methanol to block the endogenous peroxidase activity, and rinsed with PBS. The slides were TUNEL stained using an *in situ* cell death detection POD kit (Roche, Penzberg, Germany) in accordance with the manufacturer's instructions. All slides were counterstained with hematoxylin. As a negative control, the terminal transferase was omitted. 100 cells were successively counted for each field by an observer who did not identify the slides. The ratio of TUNEL-positive cell number to the total cell number is shown.

### 2.5. Western Blot

After sodium dodecyl sulfate polyacrylamide gel electrophoresis (SDS-PAGE), the proteins (20 *μ*g/lane) were electrophoretically transferred onto a nitrocellulose membrane (Whatman, London, UK), which was blocked with nonfat dry milk in buffer. The membrane was incubated with primary antibodies against caspase-3, caspase-9, bax, and bcl-2 antibody (Santa Cruz Biotechnology, Santa Cruz, CA, USA) and second antibody goat anti-mouse IgG conjugated with horseradish peroxidase (Santa Cruz Biotechnology). Thereafter, the proteins were visualized by an electrochemiluminescence detection system (GE Healthcare Bio-Sciences, Uppsala, Sweden) and analyzed by Quantity One Analysis Software (Bio-Rad Laboratories, Hercules, CA, USA). *β*-actin was used as protein loading control. 

### 2.6. Statistical Analysis

All data are expressed as mean ± SEM. Statistical analysis was performed using the paired Student's *t*-test of the SPSS 10.0 statistical software package (SPSS, Chicago, IL, USA). The threshold of significance was defined as *P* < 0.05.

## 3. Results and Discussion

### 3.1. MiR-210 Expression

**Figure 1:**
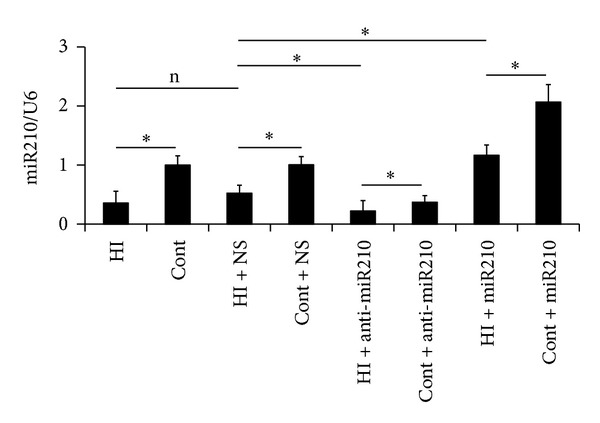
MiR-210 expression. MiR-210 expression was downregulated in the brain 72 h after HI injury (HI) than in normal control (Cont). MiR-210 was downregulated in the brain of rats intraventricular injected with miR-210 inhibitor (anti-miR210), while upregulated in the brain of rats intraventricular injected with miR210 mimic (miR-210). Our data showed that values shown were the means ± SD of three independent experiments performed in triplicate (*P < 0.05).

We confirmed the expression level of miR-210 using quantitative real-time PCR analysis. U6 was used as the endogenous control because it was the most stably expressed miR across all subjects in the control and experimental groups. MiR-210 was robustly downregulated in the brain of rats intraventricular injected with miR-210 inhibitor, while upregulated in the brain of rats intraventricular injected with miR-210 minic, which confirmed that animal models were prepared successfully (Figure 1). 

Although miR-210 was reported to be upregulated in both normal and transformed hypoxic cells [3, 6, 12], mouse models of brain transient focal ischaemia [4] and cardiac hypertrophy/cardiac failure, and in placentas from patients with preeclampsia, our data showed that miR-210 expression was downregulated in the brain 72 h after HI injury than in normal control (Figure 1). Also, Liu et al. reported that miR-210 was downregulated in both brain and blood 24 h after brain ischemia compared with untouched control animals [5]. 

The animal data and cell culture results suggested that miR-210 was elevated immediately in response to hypoxia and subsequently decreased several days later [6, 13]. It has been shown that there are more persistent alterations of HIF-1*α* expression when hypoxia is accompanied by ischemia. In a postnatal 12 d rat HI model, van den Tweel et al. reported that the level of HIF-1*α* protein increased and peaked at 3 h but returned to baseline at 6 h after injury [14]. In 10 d pups after 1.5 h middle cerebral artery (MCA) occlusion, HIF-1*α* protein peaks at 8 h and declines subsequently at 24 h in the injured cortex [15]. So, the level of miR-210, which is robustly upregulated by HIF-1*α*, is dependent on the duration after HI injury. Additional investigations are needed to demonstrate the differential expression of miR-210 in the brain in different time points after HI injury. 

### 3.2. Effects of MiR-210 on Neuronal Apoptosis and Protein Expressions of Caspase-3, Caspase-9, Bax, and bcl-2

**Figure 2:**
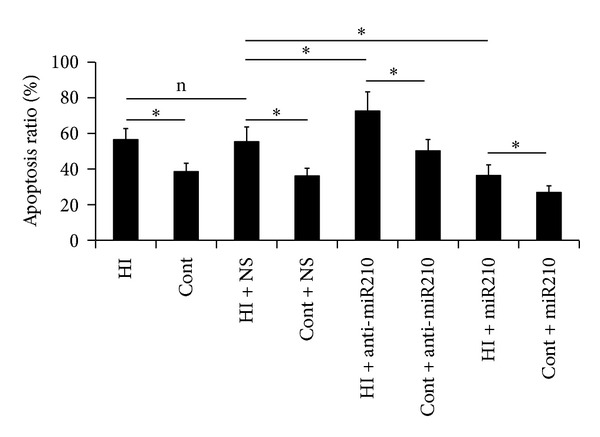
Effects of miR-210 on neuronal apoptosis. Apoptosis in brain sections was investigated by TUNEL assay. The percentage of TUNEL-positive cells increased in all HI groups (HI) compared with the control group (Cont). The ratio of TUNEL-positive cells in miR-210 overexpression rats (miR-210) decreased, while increased obviously in miR-210 block rats (anti-miR-210). Values shown were the means ± SD of three independent experiments performed in triplicate (*P < 0.05).

**Figure 3:**
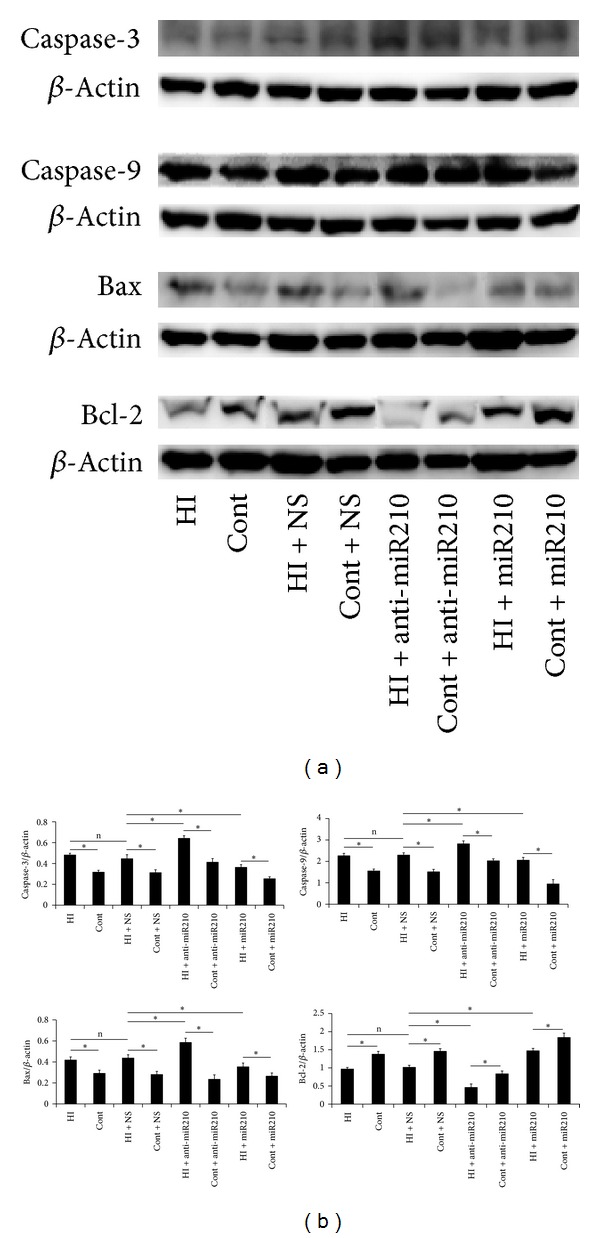
Effects of miR-210 on protein expressions of caspase-3, caspase-9, bax, and bcl-2. Caspase-3, caspase-9, and bax protein levels were enhanced in miR-210 block rats (anti-miR-210) and decreased in miR-210 overexpression rats (miR-210) compared to controls (Cont). In contrast, antiapoptotic bcl-2 expression was decreased in miR-210 block mice (anti-miR-210) and increased in miR-210 overexpression mice (miR-210) compared to controls (Cont). (a) Density values shown were the means ± SD of three independent experiments performed in triplicate (*P < 0.05) (b).

Apoptosis in brain sections was investigated by TUNEL assay. The percentage of TUNEL-positive cells was remarkably increased in all HI groups compared with the control group. There were few TUNEL-positive cells in miR-210 overexpression rats. By contrast, the ratio of TUNEL-positive cells increased obviously in miR-210 block rats (Figure 2). We next assessed the effects of miR-210 on apoptosis-related protein expression. Western blot analysis demonstrated that caspase-3, caspase-9, and bax protein levels were enhanced in miR-210 block rats and decreased in miR-210 overexpression rats compared to controls. In contrast, antiapoptotic bcl-2 expression behaved in an almost inverse manner (Figure 3). 

Previous studies have shown that miR-210 can protect cells from hypoxia-induced apoptosis [6, 10, 16]. Conversely, miR-210 blockade in the presence of hypoxia induces apoptosis [6]. Likewise, our data also revealed that miR-210 in the presence of hypoxia can prevent cell apoptosis. Apoptosis involves a series of gene activation, expression, and regulation events, and it plays an important role in HI brain injury by acting as an important form of delayed neuronal death [17]. Western blot analysis demonstrated that caspase-3, caspase-9 and bax protein levels decreased and bcl-2 expression increased in miR-210 overexpression rats, suggesting that miR-210 suppressed neuronal apoptosis by inhibiting caspase activity and regulating the balance between bcl-2 and bax levels. These pieces of evidence suggested that miR-210 has a neuroprotection and restoration feature by suppressing neuronal apoptosis. The lower miR-210 levels at 72 h after HI injury suggested that the repair function of miR-210 was diminished at late stage after neonatal HI injury.

## 4. Conclusions

The blood-brain barrier (BBB) has been shown to be more permeable to various blood-borne solutes and small lipid-insoluble molecules in the fetal rat brain than in adults. The length of miRs is only 18–25 nts and readily crosses the BBB to the HI area through vector [18, 19]. So, miR-210 delivery through circulation may work as a novel therapeutic intervention for treatment of HIE. Further investigations of its regulation, its targets, and its physiological and/or pathogenic effects in HIE are eagerly required in the future.
